# 

**Published:** 1906-11

**Authors:** 


### CONTENTS.

ORIGINAL COMMUNICATIONS—

MEDICAL SOCIOLOGY IN RELATION TO MEDICAL COLLEGES AND THE PROFESSION. By R. R. Kime, M.D., Atlanta, Ga ........ 505

RADIOTHERAPY IN MALIGNANT DISEASE IN THE NEIGHBORHOOD OF THE EYE. By Bernard Wolff, M.D., Atlanta, Ga ........ 511

TUBERCULOSIS. By J. W. Hurt, M.D., Atlanta, Ga ........ 514

CALCIUM SULPHIDE AS A PROPHYLACTIC. By A. T. Cuzner, M.D., Gilmore, Fla ........ 519

EDITORIAL NOTES AND COMMENTS—

THE CURE OF THE SOCIAL EVIL ........ 522

AGAIN THE GREAT AMERICAN FRAUD ........ 524

NEWS AND NOTES ........ 526

CONTENTS CONNTINNUED ........ X

